# Spatial Distribution of DARPP-32 in Dendritic Spines

**DOI:** 10.1371/journal.pone.0075155

**Published:** 2013-09-10

**Authors:** Hans Blom, Daniel Rönnlund, Lena Scott, Linda Westin, Jerker Widengren, Anita Aperia, Hjalmar Brismar

**Affiliations:** 1 Science for Life Laboratory, Stockholm, Sweden; 2 Department of Applied Physics, Royal Institute of Technology, Stockholm, Sweden; 3 Department of Women’s and Children’s Health, Solna, Sweden; Institute for Interdisciplinary Neuroscience, France

## Abstract

The phosphoprotein DARPP-32 (dopamine and cyclic adenosine 3´, 5´-monophosphate-regulated phosphoprotein, 32 kDa) is an important component in the molecular regulation of postsynaptic signaling in neostriatum. Despite the importance of this phosphoprotein, there is as yet little known about the nanoscale distribution of DARPP-32. In this study we applied superresolution stimulated emission depletion microscopy (STED) to assess the expression and distribution of DARPP-32 in striatal neurons. Primary culture of striatal neurons were immunofluorescently labeled for DARPP-32 with Alexa-594 and for the dopamine D1 receptor (D1R) with atto-647N. Dual-color STED microscopy revealed discrete localizations of DARPP-32 and D1R in the spine structure, with clustered distributions in both head and neck. Dissected spine structures reveal that the DARPP-32 signal rarely overlapped with the D1R signal. The D1R receptor is positioned in an “aggregated” manner primarily in the spine head and to some extent in the neck, while DARPP-32 forms several neighboring small nanoclusters spanning the whole spine structure. The DARPP-32 clusters have a mean size of 52 +/- 6 nm, which is close to the resolution limit of the microscope and corresponds to the physical size of a few individual phosphoprotein immunocomplexes. Dissection of synaptic proteins using superresolution microscopy gives possibilities to reveal in better detail biologically relevant information, as compared to diffraction-limited microscopy. In this work, the dissected postsynaptic topology of the DARPP-32 phosphoprotein provides strong evidence for a compartmentalized and confined distribution in dendritic spines. The protein topology and the relatively low copy number of phosphoprotein provides a conception of DARPP-32’s possibilities to fine-tune the regulation of synaptic signaling, which should have an impact on the performance of the neuronal circuits in which it is expressed.

## Introduction

Communication between nerve cells in the brain can simplistically be described as a biochemical concert of synaptic neurotransmitters, receptors, ion channels and effector molecules, coding and controlling signal transmission. Regulation of signaling efficiency is basically controlled by down-stream (and up-stream) regulating molecular system that modulates synaptic transmission. To elucidate molecular mechanisms in the finest structures of the nervous system dissecting synaptic assemblies is thus of large interest in neurobiology [1].

In the case of the neurotransmitter dopamine, which plays a central role in reward-driven processes and motor activity, down-stream effects are mediated *via* interaction with G protein coupled receptors (e.g. D1- and D2-like), secondary messengers (e.g. cAMP, Ca^2+^) and different effector molecules [2]. An important effector molecule in the dopaminergic signaling pathway, mediating the action of dopamine, is the dopamine- and cAMP-regulated phosphoprotein of 32 kDa (DARPP-32) [3]. This phosphoprotein is expressed primarily in medium-sized spiny neurons of the neostriatum [4], which receive dopaminergic as well as glutamatergic stimulation of connecting neurons from the midbrain, cortex and thalamus.

Accumulated evidence collected during the last decades have shown that DARPP-32 is a key modulator of numerous transduction cascades [5,6]. The phosphoprotein regulates the efficacy of transduction by acting as a potent substrate for several kinases and phosphatases. The regulated enzymatic activities modulate and control synaptic conductance by mediating changed phosphorylation/dephosphorylation levels of neuronal receptors, ion channels and ion pumps [2,3]. DARPP-32’s broad functional behavior is achieved by different phosphorylation sites on the cytosolic phosphoprotein [5,6].

Several tissue and cell specific studies of the distribution of DARPP-32 in the neostriatum have been done during the last decades [7–10]. Despite the importance of this key phosphoprotein, there is as yet little known about the postsynaptic distribution of DARPP-32. In this study we have applied the novel superresolution stimulated emission depletion microscopy (STED) technique to assess how DARPP-32 is expressed and distributed. The achieved nanoscale resolution reveals that the phosphoprotein is compartmentalized and confined in the postsynaptic region of dendritic spines in striatal neurons.

## Results

The postsynaptic localization of DARPP-32 in dendritic spines was studied in cultured striatal neurons (derived from E18.5 Sprague dawley rat embryos). Cells were maintained in culture for 3 weeks before imaging experiments (three separate cultures from three embryos of different litter were used). Imaged dendritic spines were all located on secondary dendritic branches connected to main dendrites attached to the soma. Immunofluorescently labeled neurons showed rich dendritic branching (density of up to 1 spine/µm) with spines being mushroom shaped, thin or stubby, as shown previously in striatal cultures [11,12].

**Figure 1:**
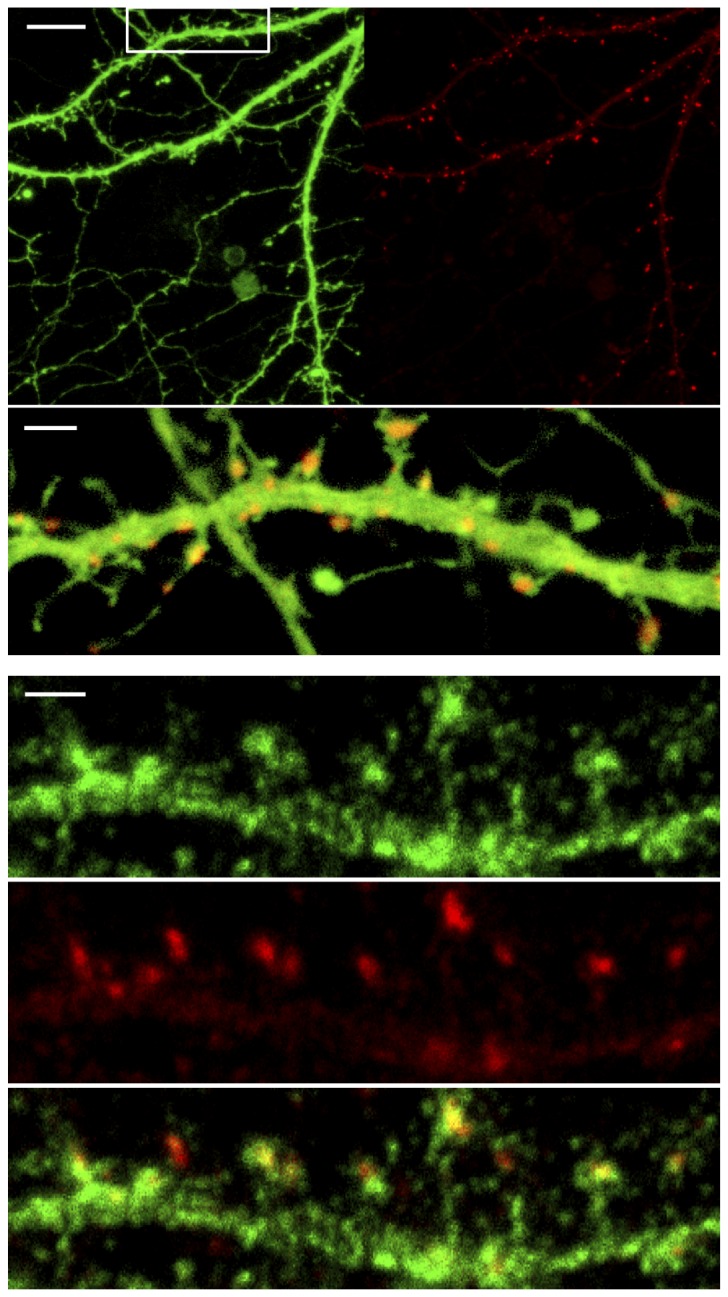
Striatal cultured neurons. Confocal overview of striatal cultures showing dendritic spine structure morphology (*Upper*). EGFP filled neurons in green; PSD-95-mCherry in red, including a zoomed in overlay along the dendrite shown in the white rectangle (*Lower*). Striatal neurons immunolabeled with Darpp-32 in green and PSD-95 in red. Scale bars: 5 µm and 1 µm (*upper*) and 1 µm (*lower*).

Figure 1 shows an overview of the dendritic morphology where striatal neurons were transfected with EGFP filling the neurons (green), and PSD-95-mCherry (red), as well as coimmunolabeling for Darpp-32 (green) and the synaptic scaffolding protein PSD-95 (red). Due to the diffraction limit of light, classical fluorescence microscopy cannot resolve the postsynaptic distribution of DARPP-32 within a single spine. To overcome this inherent problem, we applied superresolution STED microscopy to dissect the nanoscale topology of immunofluorescently labeled DARPP-32. In essence, the STED technique shrinks a conventional diffraction-limited focal spot by switching off neighboring fluorescent molecules sequentially, thus allowing nanoscale images to be generated [13].

**Figure 2:**
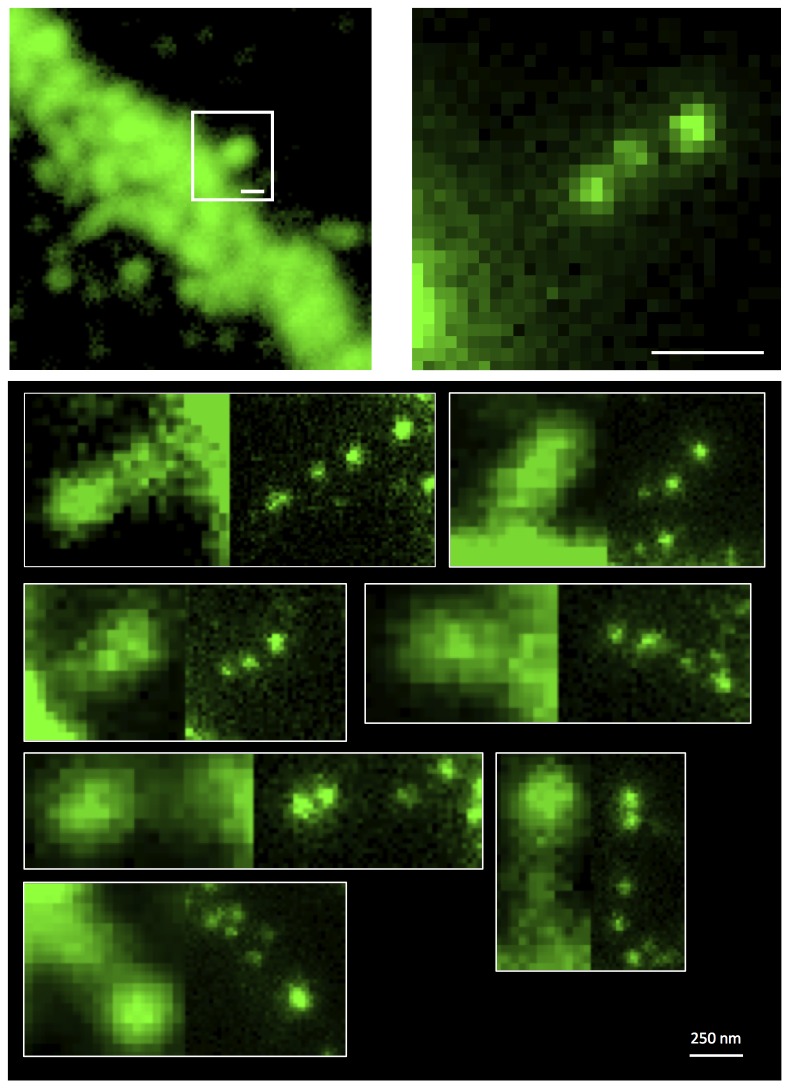
Superresolution imaging of DARPP-32. Confocal microscopy image showing an overview of a striatal dendritic structure with a selected spine (*Upper left* – green; squared box). Distribution of immunolabeled DARPP-32 imaged with superresolution STED microscopy (*Upper right* - green). (*Lower*) Gallery of dendritic spine and their DARPP-32 distributions as resolved with confocal microscopy and STED microscopy. All images are raw, unprocessed data. Scale bars 250 nm.

**Figure 3:**
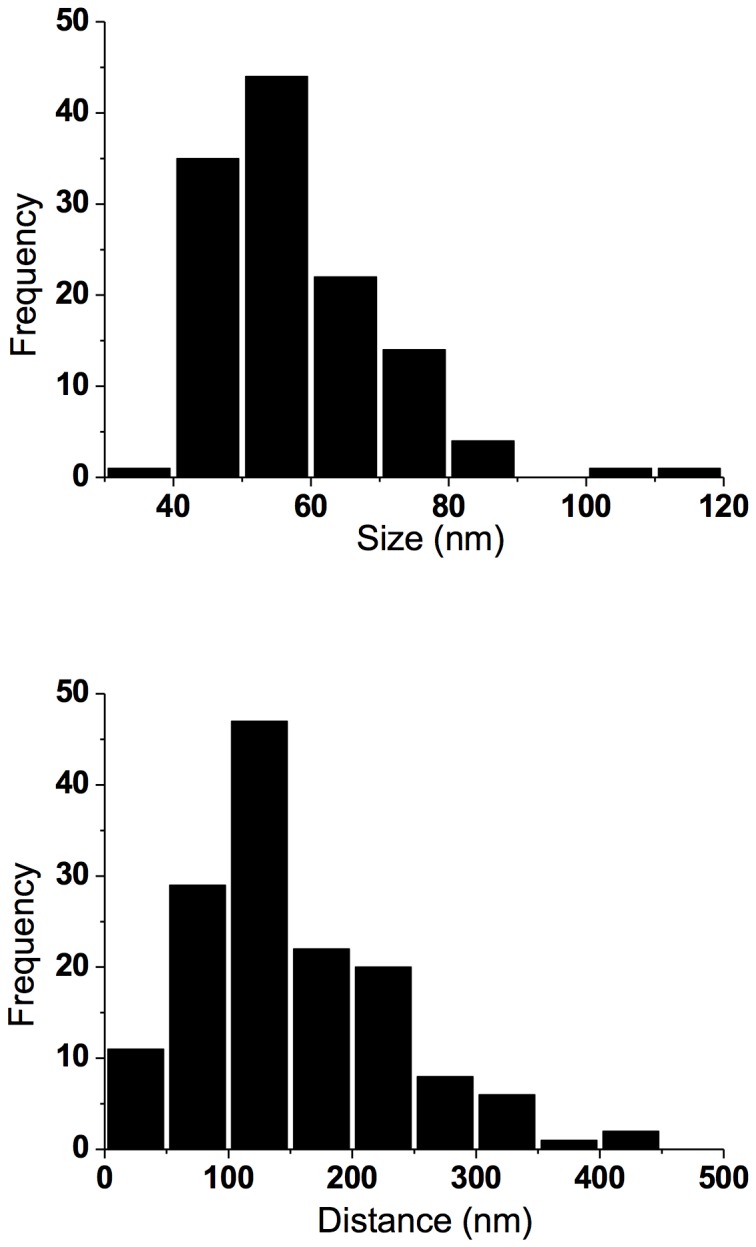
Spatial topology of DARPP-32. (*Upper*) Histogram showing the spatial extent of DARPP-32 nanoclusters in spines (n = 20), mean cluster size 52 +/- 6 nm (*Lower*). Histogram showing the cluster-to-cluster distances of DARPP-32 in spines (n = 20), mean distance between phosphoprotein clusters 110 +/- 40 nm.

As is seen in Figure 2, conventional confocal microscopy does not allow resolving the nanoscale topology of DARPP-32 in dendritic spines. One may instead be misled to assume that the spines are filled with the phosophoprotein. With STED microscopy, clusters of DARPP-32 are however dissected and localized within the spine head and the spine neck (mean cluster size 52 +/- 6 nm, cf. Figure 3). The minimum size of these clusters is around 40 nm, which reflects the physical size of a few immunocomplexes (phosophoprotein + primary + secondary antibodies) spanning the nanoclusters. This size is thus the physical resolution our STED microscope dissect (previously also shown by imaging postsynaptic assemblies of the Na^+^,K^+^-ATPase and dopamine D1 receptor in striatal neurons [11,12]).

The imaged gallery of dendritic spines in Figure 2 shows a heterogeneous distribution of the phosphoprotein. Analyzing the fluorescence cluster intensity of DARPP-32, and comparing it to individual spots of single antibodies (unspecifically) attached to the bare cover glass, only a four times higher intensity is deduced for the DARPP-32 cluster (mean peak intensity of single spots = 32 ± 12 counts, *n* = 13; mean peak intensity of DARPP-32 spots = 121 ± 10 counts, *n* = 20). This estimated brightness-ratio indicates that only a handful of phosphoprotein complexes are located (labeled) in individual nanoclusters, and furthermore each spine visualized seems to contain only a couple of DARPP-32 clusters. The latter is quantitatively analyzed by a cluster-to-cluster distance analysis, which indicates a spatially sparse population of endogenous DARPP-32 in the spine structures (cf. Figure 3; mean distance between phosphoprotein clusters 110 +/- 40 nm).

**Figure 4:**
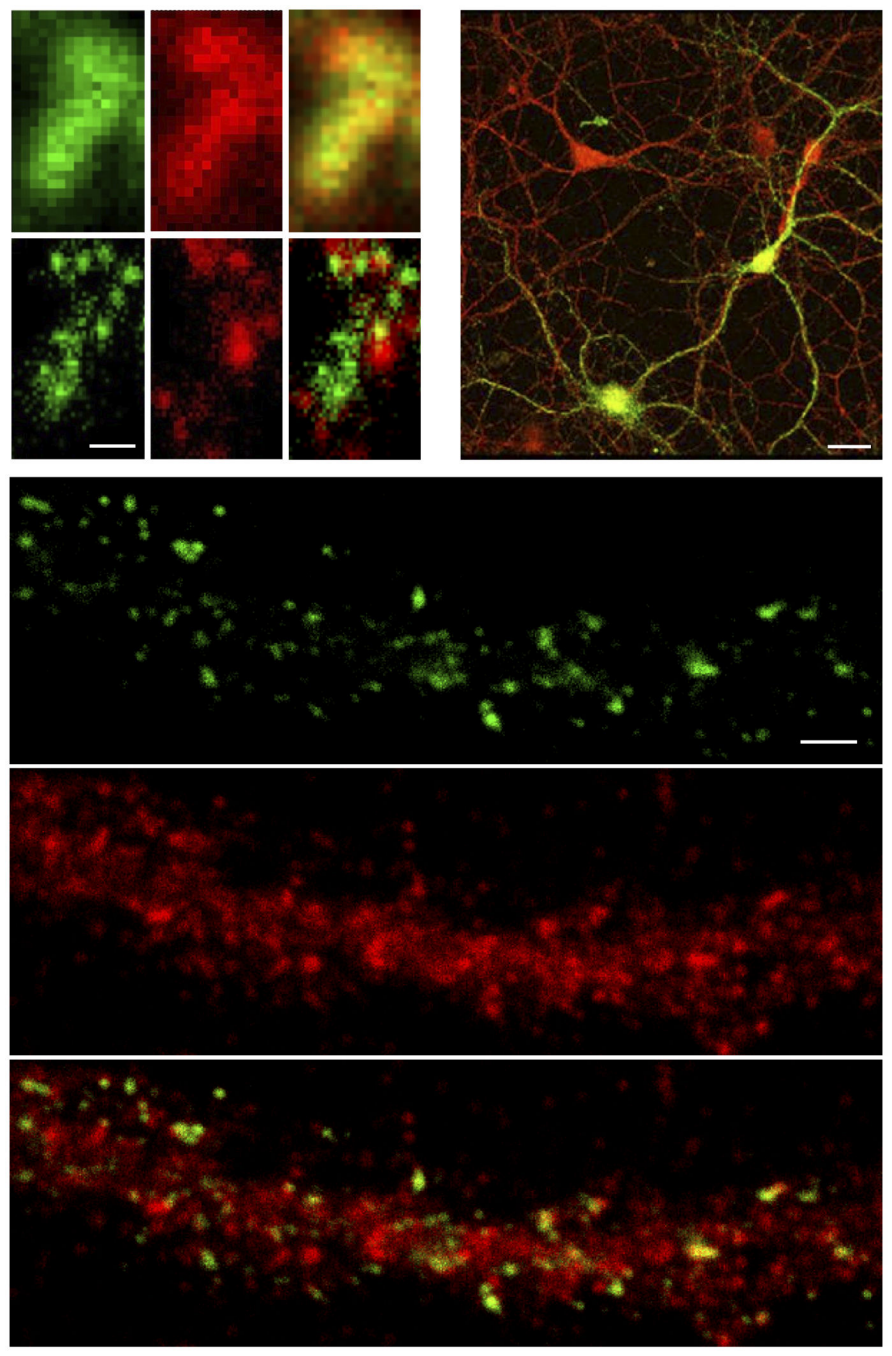
DARPP-32 in dopaminergic neurons. (*Upper left*) Confocal and corresponding STED microscopy images of DARPP-32 (*green*) and dopamine 1 receptor (*red*) in the spine structure. Scale bar 250 nm. All images are raw, unprocessed data (*Upper right*). Over-view of cultured striatal cells immunolabeled for DARPP-32 (*green*) and the dopamine 1 receptor (*red*) imaged with a Leica SP5 confocal microscope. Scale bar 20 µm (*Lower*). Striatal neurons immunolabeled with D1R in green and PSD-95 in red. Scale bar 1 µm.

**Figure 5:**
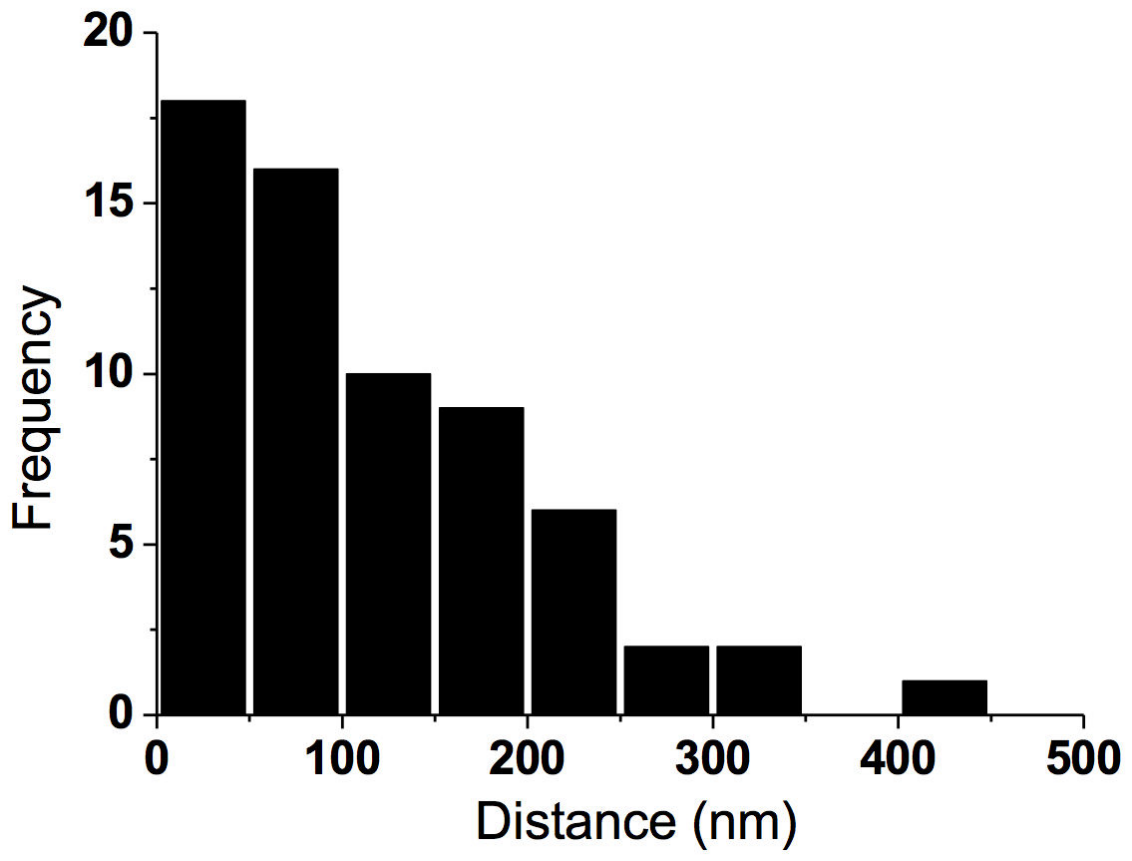
Postsynaptic occurrence of DARPP-32 and D1R. Histogram showing the cluster-to-cluster distances of DARPP-32 to D1R in spines (n = 8), mean distance between clusters 70 +/- 40 nm.

Figure 4 shows dual-color images of dendritic spines containing DARPP-32 and the dopamine 1 receptor (D1R). Confocal microscopy indicates a large co-localization of D1R and DARPP-32 (yellow overlap). In the STED images both compartmentalized DARPP-32 (green) and D1R (red) clusters are dissected in the dendritic spines, where nanosized pools of the receptor and phosphoprotein are neighboring as well as “on-top” of each other. The co-occurrence of receptor with the synaptic scaffolding protein PSD-95 indicates that both the receptor and phosphoprotein are postsynaptically expressed (shown with biochemistry in [12]). Basically the receptor seems to be positioned in an “aggregated” manner in the spine head and the neck, as shown previously [12]. DARPP-32 again shows a handful of nanoclusters distributed over the spine, and the nearest neighbor distance to the D1R receptor has a mean of 70 +/- 40 nm (cf. Figure 5).

## Discussion

Regulation of postsynaptic signal transmission in medium spiny neurons in the striatum is mediated via a cascade of biochemical reactions. Important for controlling and fine-tuning transmission, as shown during the last decades, is DARPP-32, a signaling integrator and hub molecule that effectively modulate the properties of the neuronal circuit [2,3,5,6]. Here we show with superresolution STED microscopy the postsynaptic expression of DARPP-32. The dissected topology of the DARPP-32 phosphoprotein provides strong evidence for a compartmentalized and confined distribution in dendritic spines. The relatively low copy number of phosphoproteins provides a conception of DARPP-32’s possibilities to fine-tune the regulation of synaptic signaling

### Amount

It has been shown in the literature that the concentrations of DARPP-32 in striatal tissue may reach such high values as 50 µM [2,3]. If this concentration is distributed homogenously it means that several hundreds or thousands of DARPP-32 phosophoprotein could occupy a dendritic spine. Mushroom shaped spines dissected in this study have a ‘confocal’ head diameter of around D~300 nm and a neck length of about L~300 nm (with an assumed neck-width of 100 nm) all in agreement with ultrastructural analysis of dendritic spines [14,15]. Applying these numbers generates a spine volume of V~2x10^-17^ L, which by multiplying with the assumed tissue concentration (50 µM) and Avogadro’s number (6.022 x 10^-23^) yields an estimate that approximately 600 copies of DARPP-32 occupies an individual dendritic spine. Note that this number might actually be higher or lower, as the morphology of the spine is only indirectly inferred from the confocal (blurred) images with the cytosolic phosphoprotein (cf. Figures 1 and 2). Immunofluorescence imaging of the membrane bound dopamine 1 receptor generates the same (un-resolved) confocal extent of the dendritic spine (cf. Figure 4). In essence this basically tells us that in order to study the finest structure of the nervous system, superresolution fluorescence microscopy is a more suitable tool [16].

The estimated amount of the phosphoprotein deduced above seems to be somewhat higher than what is concluded from the STED images shown in this study. From the superresolution images, estimates of tens of DARPP-32 molecules in individual spines are deduced, meaning just a few micromoles in concentration. In the literature is has actually been pointed out that the concentration of DARPP-32, compartmentalized in spines, is most probably not as high as 50 µM [6]. *In silico* mathematical modeling and simulations of how the quantity of DARPP-32 in dendritic spines influence signal integration indicate that the system is very robust [17]. In the work by Fernandez et al. sub-micromolar concentrations of the phosphoprotein were able to swiftly regulate neuronal signaling (temporal relationship between cAMP and Ca^2+^ from dopamine and glutamate signals) in medium-sized spiny neurons. Estimates of tens of DARPP-32 regulating molecules in mushroomed shaped spines, as seen in this study, could thus be enough for modulating postsynaptic neuronal circuit properties.

Furthermore, as shown in the literature, the median inhibitory concentration of phosphorylated DARPP-32 for inhibition of protein phosphatase-1 is in the nanomolar range [3]. This means that just a few DARPP-32 molecules might be enough to tune the signal transduction in the dendritic spine. Such small numbers intuitively seems to be extreme on the macro scale; however, the microscopic size of the compartmentalized and crowded spine basically fits (or need) only a small number of synaptic proteins (receptors, channels or pumps) [18].

### Topology

In the work by Oliveira et al. [19], mathematical modeling shows that the subcellular location (formation of microdomains in the spine head or the dendrite) of adenylate cyclase-D1R complexes and protein kinase A (PKA) influences the biochemical signaling in striatal neurons. The heterogeneous distribution of DARPP-32, as revealed in this study, shows a topology that thus might be important for the performance of the neuronal circuits. Larger clusters of the phosphoprotein are more frequently seen in the head area. DARPP-32 clusters located in the spine head might indicate a population involved in fine-tuning the synaptic transmission. Additional DARPP-32 clusters located along the neck could then speculatively be a reserve pool for changed synaptic activity. However, a more realistic assumption is that the pools of phosphoprotein in the neck regulate other properties within the spine structure, as DARPP-32 is the hub of a rich network of regulations [5,6]. The neck population might additionally allow the spine to function as a discreet chemical compartment, regulating and isolating concentration dynamics of ions and intracellular messenger molecules to individual spines [20].

### Co-occurrence

Regarding the nanoscale distributions of the phosphoprotein and the dopamine 1 receptor, classical imaging over-estimates any co-localization due to limited optical resolution. Membrane bound D1R and cytosolic DARPP-32 is in essence artificially merged into the same blurred spot. With superresolution imaging there is however only a very small overlap between the receptor and the phosphoprotein (cf. Figures 4 and 5). This overlap may in turn be somewhat ‘induced’ as the resolution of our STED microscope is not infinite; our 40 nm maximum resolution in the focal plane may thus slightly merge D1R and DARPP-32 as well. On the other hand the immunolabeling system (receptor/phosophoprotein + primary + secondary antibodies) may separate D1R and DARPP-32 clusters about 15-20 nanometer. The latter is in large not well resolved (shift of green and red nanoclusters), but are influences that should always be kept in mind when interpreting superresolution images [21].

Moreover, knowing that the phosphoprotein is a signaling hub-molecule (a substrate for several kinases and phosphatases) that participates in postsynaptic regulation of several neurotransmitter systems [5,6], a small overlap with D1R is expected and this basically confirms DARPP-32’s biochemically important roles. The small overlap of D1R and DARPP-32 probably points toward a biochemical and not a direct interaction between the cytosolic phosphoprotein and the membrane bound receptor. As DARPP-32 is a phosphorylation-dependent substrate, one would assume that only with dopamine stimulation might DARPP-32 be co-localized with D1R to a larger extent. Under basal condition the phosphoprotein is not phosphorylated and thus not bound to protein phosphatase-1 that can be localized in the vicinity of the synaptic area [3]. A nanoscale separation (i.e. very low co-occurrence) of receptor and phosphoprotein is thus very plausible, which is also found in this study.

Compared to previous investigations using confocal microscopy of tissue [22–24], less co-occurrence of DARPP-32 and D1R was found in individual neurons (~25% are simultaneously D1R and DARPP-32 positive; > 60% are just D1R positive, while less than 15% stains for DARPP-32 only). This discrepancy is likely due to that the distribution of DARPP-32 and D1R in cultured neurons, is not fully mapping the ‘mature’ distribution in tissue. A somewhat higher abundance of phosphoprotein could thus be the case *in vivo*.

## Conclusion

Deciphering the synaptic biochemical machinery in the brain, and link its molecular orchestra to physiological processes like memory, behavior and psychiatric dysfunctions is a grand challenge in neuroscience. Application of superresolution imaging prompts to give us help to elucidate some of the underlying questions. The benefit of a better resolved context will give improved support to current hypotheses but also reveal unexpected new findings. In this study, the dissected distribution of the signal integrator molecule DARPP-32 reveals a discrete localization of the phosphoprotein in the postsynaptic structures in striatal neurons. In essence, the resolved topology of the phosphoprotein provides a nanoscale still-image in support of the assumed central role of DARPP-32. The results point toward a heterogeneous confinement of DARPP-32 slightly enriched in the head, possibly fine-tuning synaptic properties, with additional pools in the neck that modulate transmission processes or other properties in the chemically confined spine structure. The relatively low abundance of the phosphoprotein, as resolved by superresolution STED imaging, further suggests that postsynaptic performance can be modulated even using very few copies of the phosphoprotein. Moreover, the small amount of co-occurrence with the dopamine 1 receptor indicates that DARPP-32 participate in regulation of additional postsynaptic signaling systems in dopaminergic neurons.

## Methods

### Ethics statement

All animals used in this study were obtained from Scanbur AB, Sollentuna, Sweden. All animal procedures were performed according to the Karolinska Institutet regulations concerning care and use of laboratory animals and approved by the local ethical committee (Stockholm North ethical evaluation board for animal research, applications N35/08 and N60/11). All efforts were made to minimize the number of animals used and their suffering.

### Cell culture

Pregnant 18.5 days SD rat females were anesthetized using CO_2_, embryos were removed and females sacrificed by severing the aorta. Embryos were decapitated and striatum was dissected. Dissected tissue was incubated for 10 min at 37^o^C in Hanks’ balanced salt solution Gibco Invitrogen) containing 20 mM HEPES (Sigma) and 0.25% Trypsin (Gibco Invitrogen) and dissociated in MEM (Gibco Invitrogen) by mechanical triturating using a fire polished Pasteur pipette. The cells were plated at a density of 0.8 * 10^5^ cells on round 18 mm (No. 1.5) polyornithine (Gibco Invitrogen, 80 µg/mL overnight at 37^o^C) coated coverslips and incubated for 3 h in MEM media containing 10% horse serum, 2 mM L-glutamine and 1 mM NaPyruvate. The cells where then cultured in neurobasal media (Gibco Invitrogen) containing 1X B27 (Gibco Invitrogen) and 2 mM L-glutamine and 1% penicillin/streptomycin. Cells were maintained in culture for 3 weeks before experiments and half the culture media volume was changed twice a week. The use of cultured striatal embryonic cells with maturing spine growth is a well-established system. The integrity and normal activity in the cultures were tested by imaging spontaneous activity [25]. The density of spines is typically on the order of a single spine/μm in our cultures. The shape of spines is similar to what is shown in co-culture systems that may provide a higher density [26].

### Transfection

After 3 weeks in culture, primary striatal neurons were cotransfected with green fluorescent protein (EGFP) and mCherry fused to postsynaptic density protein 95 (PSD-95-mCherry) using Lipofectamine 2000 (Invitrogen) according to the manufacturer’s recommendations. 48 hours after transfection, cells were fixated with 4% paraformaldehyde and mounted in Prolong Gold antifade reagent without DAPI (Invitrogen).

### Immunostaining

Primary cultured striatal neurons were fixated for 15 min using 4% paraformaldehyde and 4% sucrose in PBS, permeabilized using 0.1-0.2% Triton X-100 (Sigma Aldrich) in PBS and blocked with normal goat serum (NGS, Jackson ImmunoReasearch Laboratory Inc.). Cells were subsequently incubated with primary antibody for 1.5 hours at room temperature or overnight at +4°C in PBS containing NGS. The primary antibodies used were anti-DARPP-32 (1:10 000), anti-D1R (1:300) and anti-PSD-95 (1:500). Following incubation the cells were washed in PBS and then incubated at room temperature for 1-2 hours with fluorescent secondary antibody in PBS containing NGS. Cells were washed and mounted using Immu-Mount (Thermo scientific) or Prolong Gold antifade ragent without DAPI. The experimental procedure was repeated at least three times.

### Antibodies

The following primary antibodies were used for immunocytochemistry; anti-Dopamine D1 receptor rabbit polyclonal antibody, previously characterized [27], anti-DARPP-32 mouse monoclonal antibody (kind gift from Prof. Angus Nairn’s lab, Yale University, USA) and anti-PSD-95 mouse monoclonal, (Abcam ab2723), The secondary antibodies used were; Atto647N goat anti-rabbit (1:400, Sigma Aldrich), Alexa-594 goat anti-mouse (1:500, Molecular Probes Inc.) and STAR 635 goat anti-rabbit (1:200, Abberior). For colabeling experiments of PSD-95 and D1R listed antibodies was used. For colabeling experiments of PSD-95 and DARP-32, a PSD-95-Alexa 488 conjugation was selected (Alexa Fluor 488 antibody labeling kit, Invitrogen) and sequentially labeled by (1) incubation with anti-DARP-32, (2) incubation with Alexa-594 goat anti-mouse and (3) incubation with PSD-95-Alexa 488.

### STED microscopy

The system was built around a white-light super-continuum laser source (SC-450 HP, Fianium, Southampton, UK) delivering all excitation and stimulated emission depletion wavelengths. Selected wavelengths (exc1=567 nm, exc2=640 nm, STED1=710/20 nm, STED2=740/20 nm) were coupled together using a dichroic mirror (z690SPRDC, Chroma Technology Corp., Bellow Falls, US) before being sent into the microscope objective (PL APO 100×/1.40–0.7 Oil, Leica Microsystems, Wetzlar, Germany). The sample was placed on a 3D scanning piezo stage coupled to a closed loop controller unit (MAX311/M and BPC203, Thorlabs Sweden AB, Göteborg, Sweden) offering a positional resolution of 5 nm. The fluorescence from the labeled sample is collected back through the objective and separated from the excitation and the STED beams by a customized dichroic mirror (Laseroptik, Garbsen, Germany) and bandpass filters (BPF1=600/40 nm, BPF2=680/40 nm). Single photon sensitive avalanche photo-diodes (SPCMAQRH- 13-FC, PerkinElmer, Vaudreuil, Québec, Canada) fitted with multimode optical fibers (62.5 µm / 0.27 NA, M31L01, Thorlabs) acting as pinhole of 1.2–1.3 times the size of an Airy disc collected and detected the fluorescence. STED images sized 5 µm x 5 µm to 10 µm x 10 µm were acquired with a pixel size of 20 nm and a pixel dwell time of 1 ms. Average excitation powers applied were 0.4-1.0 µW and the applied STED powers were 1.6-2.4 mW (1 MHz repletion rate, ~100 ps pulse width) [28].

### Image analysis

Image analysis was performed by custom written code in Matlab (MathWorks Inc, Massachusetts, USA) where the location of each labeled protein was selected as the center of their respective emission profile. Nearest neighbor analysis was then performed to calculate the distance of each profile to the surrounding profiles of other labeled proteins, where the closest profile was selected as the nearest neighbor distance. The size of each profile was calculated as the mean full width at half maximum value measured over 20 angles. Prior to image analysis, all images were deconvoluted using 20 iterations of Richardson-Lucy algorithm and assuming a 40 nm Lorentzian shaped point spread function (PSF) [28].

## References

[1] Li Z, Sheng M (2003) Some assembly required: the development of neuronal synapses. Nat Rev Mol Cell Biol 4: 833-841. doi:10.1038/nrm1242. PubMed: 14625534. 14625534 10.1038/nrm1242

[2] Greengard P (2001) The neurobiology of slow synaptic transmission. Science 294: 1024-1030. doi:10.1126/science.294.5544.1024. PubMed: 11691979. 11691979 10.1126/science.294.5544.1024

[3] Greengard P, Allen PB, Nairn AC (1999) Beyond the dopamine receptor: the DARPP-32/Protein phosphatase-1 cascade. Neuron 23: 435-447. doi:10.1016/S0896-6273(00)80798-9. PubMed: 10433257. 10433257 10.1016/s0896-6273(00)80798-9

[4] Walaas SI, Greengard P (1984) DARPP-32, a dopamine- and adenosine 3':5'-monophosphate-regulated phosphoprotein enriched in dopamine-innervated brain regions. I. Regional and cellular distribution in the rat brain. J Neurosci 4: 84-98. PubMed: 6319627. 6319627 10.1523/JNEUROSCI.04-01-00084.1984 PMC6564747

[5] Svenningsson P, Nishi A, Fisone G, Girault JA, Nairn AC et al. (2004) Darpp32: An integrator of neurotransmission. Annu Rev Pharmacol Toxicol 44: 269-296. doi:10.1146/annurev.pharmtox.44.101802.121415. PubMed: 14744247. 14744247 10.1146/annurev.pharmtox.44.101802.121415

[6] Yger M, Girault J-A (2011) DARPP-32, jack of all trades… master of which? Frontiers in Behavioral Neuroscience 5: 1-14. 21927600 10.3389/fnbeh.2011.00056 PMC3168893

[7] Won L, Price S, Wainer BH, Hoffmann PC, Bolam JP et al. (1989) Correlated light and electron microscopic study of dopaminergic neurons and their synaptic junctions with DARPP-32-containing cells on three-dimensional reaggregate tissue culture. The journal of comparatively neurology 289: 165-177. doi:10.1002/cne.902890114. 10.1002/cne.902890114 2572612

[8] Ouimet CC, Greengard P (1990) Distribution of DARPP-32 in the basal ganglia: an electron microscopic study. J Neurocytol 19: 39-52. doi:10.1007/BF01188438. PubMed: 2191086. 2191086 10.1007/BF01188438

[9] Ouimet CC, Lamantia AS, Goldman-Rakic P, Rakic P, Greengard P (1992) Immunocytochemical localization of DARPP-32, and dopamine and cyclic-AMP-regulated phosphoprotein, in the primate brain. The journal of comparatively neurology 323: 209-218. doi:10.1002/cne.903230206. 10.1002/cne.903230206 1328330

[10] Glausier JR, Maddox M, Hemmings HC, Nairn AC, Greengard P et al. (2010) Localization of dopamine- and cAMP-regulated phosphoprotein-32 and inhibitor-1 in area 9 of Macaca mulatta prefrontal cortex. Neuroscience 167: 428-438. doi:10.1016/j.neuroscience.2010.02.014. PubMed: 20156529. 20156529 10.1016/j.neuroscience.2010.02.014 PMC2863358

[11] Blom H, Rönnlund D, Scott L, Spicarova Z, Widengren J et al. (2011) Spatial distribution of Na+,K+-ATPase in dendritic spines dissected by nanoscale superresolution STED microscopy. BMC Neurosci 12: 16. doi:10.1186/1471-2202-12-16. PubMed: 21272290. 21272290 10.1186/1471-2202-12-16 PMC3040715

[12] Blom H, Rönnlund D, Scott L, Rantanen V, Widengren J et al. (2012) Nearest neighbor analysis of dopamine D1 receptors and Na+–K+-ATPases in dendritic spines dissected by STED microscopy. Microscopy Research and Techniques 75: 220-228. doi:10.1002/jemt.21046. 10.1002/jemt.21046 21809413

[13] Hell SW (2007) Far-Field Optical Nanoscopy. Science 316: 1153-1158. doi:10.1126/science.1137395. PubMed: 17525330. 17525330 10.1126/science.1137395

[14] Wilson CJ, Groves PM, Kitai ST, Linder JC (1983) Three-dimensional structure of dendritic spines in the rat neostriatum. J Neuorsci 3: 383-398. PubMed: 6822869. 10.1523/JNEUROSCI.03-02-00383.1983 PMC6564480 6822869

[15] Rafael Y (2010) Dendritic spines. Cambridge: The MIT Press.

[16] Dani A, Huang B (2010) New resolving power for light microscopy: applications to neurobiology. Curr Opin Neurobiol 20: 648-652. doi:10.1016/j.conb.2010.07.006. PubMed: 20728340. 20728340 10.1016/j.conb.2010.07.006

[17] Fernandez E, Schiappa R, Girault JA, Le Novère N (2006) DARPP-32 is a robust integrator of dopamine and glutamate signals. PLOS Comput Biol 2: 1619-1633. PubMed: 17194217. 10.1371/journal.pcbi.0020176 PMC1761654 17194217

[18] Sheng M, Hoogenraad CC (2007) The postsynaptic architecture of excitatory synapses: a more quantitative view. Annu Rev Biochem 76: 823-847. doi:10.1146/annurev.biochem.76.060805.160029. PubMed: 17243894. 17243894 10.1146/annurev.biochem.76.060805.160029

[19] Oliveira RF, Kim MS, Blackwell KT (2012) Subcellular location of PKA controls striatal plasticity: stochastic simulations in spiny dendrites. PLOS Comput Biol 8:e1002383 PubMed: 22346744. 22346744 10.1371/journal.pcbi.1002383 PMC3276550

[20] Nimchinsky EA, Sabatini BL, Svoboda K (2002) Structure and function of dendritic spines. Annu Rev Physiol 64: 313-353. doi:10.1146/annurev.physiol.64.081501.160008. PubMed: 11826272. 11826272 10.1146/annurev.physiol.64.081501.160008

[21] Gould TJ, Hess ST, Bewersdorf J (2012) Optical nanoscopy: from acquisition to analysis. Annu Rev Biomed Eng 14: 231-254. doi:10.1146/annurev-bioeng-071811-150025. PubMed: 22559319. 22559319 10.1146/annurev-bioeng-071811-150025 PMC3568761

[22] Anderson KD, Reiner A (1991) Immunohistochemical localization of DARPP-32 in striatal projection neurons and striatal interneurons: implications for the localizations of D1-like dopamine receptors on different types of striatal neurons. Brain Res 568: 235-243. doi:10.1016/0006-8993(91)91403-N. PubMed: 1839966. 1839966 10.1016/0006-8993(91)91403-n

[23] Langley KC, Bergson C, Greengard P, Ouimet CC (1997) Co-localization of the D1 dopamine receptor in a subset of DARPP-32 containing neurons in rat caudate-putamen. Neuroscience 78: 977-983. doi:10.1016/S0306-4522(96)00583-0. PubMed: 9174066. 9174066 10.1016/s0306-4522(96)00583-0

[24] Rajput PS, Kharmate G, Somvanshi RK, Kumar U (2009) Colocalization of dopamine receptor subtypes with dopamine and cAMP-regulated phosphoproteins (DARPP-32) in rat brain. Neurosci Res 65: 53-63. doi:10.1016/j.neures.2009.09.120. PubMed: 19465068. 19465068 10.1016/j.neures.2009.05.005

[25] Aizman O, Brismar H, Uhlén P, Zettergren E, Levey AI et al. (2000) Anatomical and physiological evidence for D1 and D2 dopamine receptor colocalization in neostriatal neurons. Nat Neurosci, 3: 226-230. doi:10.1038/72929. PubMed: 10700253. 10700253 10.1038/72929

[26] Penrod RD, Kourrich S, Kearney E, Thomas MJ, Lanier LM (2011) An embryonic culture system for the investigation of striatal medium spiny neuron dendritic spine development and plasticity. J Neurosci Methods, 200: 1-13. doi:10.1016/j.jneumeth.2011.05.029. PubMed: 21672554. 21672554 10.1016/j.jneumeth.2011.05.029 PMC3148294

[27] Scott L, Kruse MS, Forssberg H, Brismar H, Greengard P et al. (2002) Selective up-regulation of dopamine D1 receptors in dendritic spines by NMDA receptor activation. Proc Natl Acad Sci U S A 99: 1661-1664. doi:10.1073/pnas.032654599. PubMed: 11818555. 11818555 10.1073/pnas.032654599 PMC122247

[28] Wildanger D, Rittweger E, Kastrup L, Hell SW (2008) STED microscopy with a supercontinuum laser source. Opt Express 16: 9614-9621. doi:10.1364/OE.16.009614. PubMed: 18575529. 18575529 10.1364/oe.16.009614

